# Bilateral neuromuscular adaptation to acute unilateral resistance exercise in healthy older adults

**DOI:** 10.1007/s11357-025-01693-8

**Published:** 2025-05-08

**Authors:** Nishadi N. Gamage, Abdulmajeed Altheyab, Yuxiao Guo, Bethan E. Phillips, George M. Opie, John G. Semmler, Philip Atherton, Mathew Piasecki

**Affiliations:** 1 Centre of Metabolism, Ageing & Physiology (COMAP), Academic Unit of Injury, Recovery & Inflammation Sciences, School of Medicine, Faculty of Medicine and Health Sciences, National Institute for Health Research (NIHR) Nottingham Biomedical Research Centre, University of Nottingham, Royal Derby Hospital Centre, Derby, DE22 3DT United Kingdom; 2 Neurophysiology of Human Movement Laboratory, Discipline of Physiology, School of Biomedicine, University of Adelaide, Adelaide, Australia; 3 Department of Occupational Therapy, College of Applied Medical Sciences, King Saud Bin Abdulaziz University for Health Sciences, Riyadh, Saudi Arabia; 4 Institute of Sports Medicine and Health, Chengdu Sport University, Chengdu, China

**Keywords:** Cross-education, Motor units, Neuromuscular, Electromyography, Ageing, Resistance exercise

## Abstract

Resistance exercise (RE) enhances functionality in older adults and has proven effective as a means of cross-education in scenarios of unilateral disuse. However, the extent to which older adults demonstrate cross-limb transfer at the motor unit (MU) level following a single bout of unilateral RE is unclear. Thirteen healthy older adults (74.9 ± 4.8 years; 5 females) underwent bilateral neuromuscular assessments pre- and post- a single bout of unilateral RE consisting of sets of 12 repetitions of leg extension of the dominant (exercise) leg, at 75% of 1 repetition maximum, performed to failure. Maximum voluntary contraction (MVC) and force steadiness (FS) were measured. Central and peripheral features of individual MU were recorded using high-density surface electromyography and intramuscular electromyography (HDs/iEMG), during contractions normalised to 25% MVC. Following unilateral RE, MVC reduced in exercise (-14.8%, *p* < 0.001) and control (-6.9%, *p* = 0.003) legs, with reduced FS performance in the exercise leg compared to the control *(p* = 0.002). MU firing rate increased during contractions normalised to 25% baseline MVC in the exercised leg (*p* < 0.05), with no adaptation in the control leg (*p* > 0.05). All iEMG recorded measures of MU potentials remained unchanged in both legs (all *p* > 0.05). Acute unilateral RE leads to bilateral MVC reduction in older males and females, demonstrating the cross-limb transfer effect. However, adaptation of MU features was only apparent in the exercised limb, and mechanisms underlying the force decline in the non-exercised limb remain uncertain.

## Introduction

Ageing is associated with a substantial decline in muscle mass, strength, and function leading to detrimental outcomes such as mobility disorders, frailty, increased fall rates, and reduction in general health status [1]. The age-related decline in neuromuscular function is partly explained by motor unit (MU) remodelling, in which MUs generally become fewer in number and larger in size [2], and this may have implications for neuromuscular control [3]. Although the age-related loss of motor neurons and muscle fibres are irreplaceable, the structure and function of the musculoskeletal system can be improved through exercise training [4]. Therefore performing exercises, particularly resistance training, is known to be effective for older adults and elicits significant enhancements in neuromuscular function [5].

Cross-education (CE) describes the widely reported effect of unilateral training conferring benefit to the non-exercised contralateral limb [6]. Although the exact mechanism of cross-limb transfer remains uncertain, it likely has cortical origins [7, 8]. This phenomenon is effective in both the upper and lower limbs [9], following stroke [10, 11] and in orthopaedic conditions [12]. Given the age-related decline in neuromotor function [13, 14] impairing functional recovery following stroke [15], and increased risk of falls and fractures among older individuals [16, 17], with the majority presenting with single-limb fractures [18], cross-limb transfer phenomenon is particularly important for older adults [19].

Exercise-induced decline in force and/or power generation due to impaired muscle activation and/or contractile function is termed performance fatigue and originates from central and peripheral sources [20]. Similar to CE, cross-limb transfer of muscle fatigue is defined as a temporary deficit in performance of the non-exercised contralateral limb following a unilateral fatiguing protocol [21, 22]. In young adults, higher-intensity fatiguing contractions are reported to generate greater non-local muscle fatigue effects compared to lower-intensity contractions, particularly in lower limbs [23].

Ageing is not a limiting factor in some aspects of cross-limb transfer [24]. However, there are several knowledge gaps regarding cross-limb transfer, including a lack of data characterising individual MU adaptations in the knee extensors (KE). Additionally, research on this phenomenon in older populations—where cross-limb transfer could be particularly valuable as an intervention—is limited, and to our knowledge, there is no available data following a single bout of unilateral KE fatiguing exercise in older individuals. Therefore, the current study aimed to evaluate the cross-limb transfer effects of acute resistance exercise (RE) on neuromuscular function, and central and peripheral features of MU adaptation in older adults. We hypothesized that both the exercised and control limbs would display reduced function, with MU firing properties altered in both, and peripheral MU features altered in the exercised limb only.

## Methods

### Ethical approval

This study was approved by the University of Nottingham Faculty of Medicine and Health Sciences Research Ethics Committee (*FHMS—390–1121*) and was conducted in accordance with the *Declaration of Helsinki*, except for registration in a database. All participants provided written informed consent.

Seventeen healthy older adults (9 females) with a mean age (± SD) of 73.8 (± 4.9) years were enrolled in this study. Participants were excluded if they had a BMI < 18 or > 35 kg/m^2^, any diagnosed neurological, musculoskeletal, cardiovascular (such as uncontrolled hypertension, recent cardiac event), metabolic disorders (such as diabetes), chronic kidney disease, malignancy (in previous 6 months), recent steroid treatment (within 6 months), respiratory diseases or if they engaged in any structured exercise training within the past 6 months. Participants were recreationally active but not involved in standard exercise programs. All participants were advised to refrain from strenuous exercises at least 48 h before the laboratory visits. Of the 17 participants who completed the study, 13 participants (5 females, 74.9 ± 4.8 years) had sufficient data quality for high-density surface electromyography (HDsEMG) analyses, therefore data are presented for these only. Of these 13, 11 participants (4 females) were included in intramuscular electromyography (iEMG) analyses due to the low yield of MUs at one or more time points.

### Experimental procedures

**Fig. 1:**
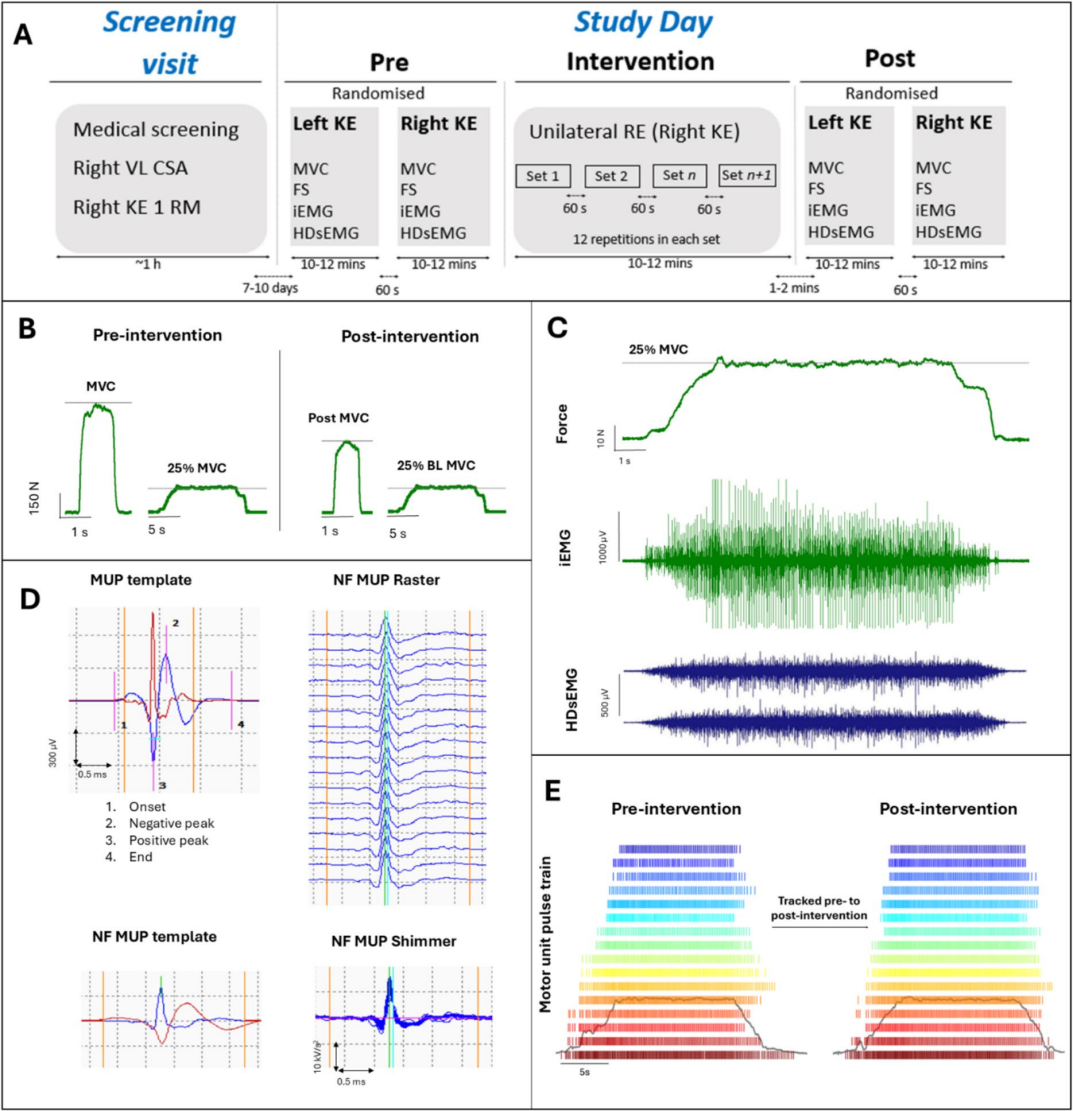
Schematic representation of the experimental protocol and timeline (**A**). Example knee extensor force traces of an older male participant showing MVC and 25% MVC of the exercise leg obtained pre- and post-RE intervention (**B**). Example force trace (upper) with corresponding intramuscular electromyography (iEMG) trace (middle) and a subset of 2 HDsEMG channels (lower) obtained during a sustained isometric knee extension at 25% of MVC (**C**). Example iEMG recorded MUP and corresponding near fibre (NF) MUP sampled with iEMG, including consecutive NFMUPs shown as raster and shimmer plots (**D**). Example HDsEMG recorded MU pulse trains (firing times) of MUs tracked from pre- to post-intervention. The grey line indicates the force trace with a plateau held at 25% MVC (**E**). *Abbreviations*: *VL *vastus lateralis*, KL *knee extensor* CSA *cross-sectional area,* 1 RM *One repetition maximum,* FS *force steadiness,* iEMG *intramuscular electromyography*, HDsEMG *High-density surface electromyography*, MVC *maximal voluntary contraction,* BL *baseline,* MUP *motor unit potential,* NF *near fibre

The experimental protocol and timeline are shown in Fig. 1A. All participants attended two laboratory visits (screening visit and study day) separated by 7–10 days.

### Screening visit

During the screening visit, participants completed a comprehensive medical screening that included fasting blood tests, electrocardiogram, blood pressure assessment, and a general health questionnaire which allowed the exclusion of participants against pre-determined criteria as listed above. Additionally, during the screening visit, 1 repetition maximum (1 RM) and VL muscle ultrasound (detailed below) were assessed.

#### One repetition maximum (1 RM) assessment

The 1 RM is defined as the maximum weight that could be lifted through the prescribed range once only [25]. A seated leg extension machine was used to measure 1 RM with an initial warm-up of 5–10 low-load repetitions performed on the dominant (right in all) limb. After one minute of rest, participants were asked to perform full knee extension movement with the load at ~ 80% of estimated 1 RM. After each successful attempt, the weight was increased progressively until a failed attempt occurred [26]. Each attempt was separated by one minute rest and 1 RM was obtained within five attempts. The 1RM was performed to enable the setting of weight for the acute RE intervention on the study day (75% of 1RM).

#### Muscle ultrasound recording

The cross-sectional area (CSA) of VL muscle of the exercise limb was measured during the screening visit using an ultrasound probe (LA523 probe, B- mode, frequency range 26- 32 Hz and MyLabTM50 scanner, Esaote, Genoa, Italy) positioned at the anatomical mid-point of VL, which was measured and identified between the greater trochanter and the midline of the patella. A conductive gel was applied to the surface of the probe to improve the fidelity of images. The ultrasound probe was positioned at the VL midpoint and moved in the medial-to-lateral direction to locate the medial and proximal borders of VL where the aponeurosis of VL intersected with the Vastus Intermedius muscle [27]. Three axial plane images were obtained and the mean area of three images was considered as CSA. Ultrasound images were analysed using ImageJ software (National Institutes of Health, United States) and the average of three ultrasound images was considered as VL CSA of the exercise limb.

### Study day assessment

Participants who completed the screening visit and were deemed eligible against the study criteria were invited to the study day assessment. Participants arrived at the laboratory at ~ 09.00 h (±1 h) after an overnight fast. The study day experimental procedures included the assessment of maximal voluntary contraction (MVC), force steadiness (FS), iEMG, and HDsEMG measures before and immediately after the unilateral RE (Fig. 1A). All described measures were individually performed on both legs and the order of testing was randomised between the legs.

### Functional properties

A purpose-built isometric dynamometer (Load cell amplifier; LCA1,12 V1 A medical PSU, GDM25B12-P1 J, Sunpower Electronics, Reading, United Kingdom) was used to measure MVC for knee extension. Participants were seated with their hip and knee flexed at approximately 90° and a seat belt was used to avoid hip or trunk movement during the leg extension. The leg to be tested was securely strapped to a plate connected to a force transducer at the ankle. After a series of moderate-intensity warm-ups, participants were instructed to perform three maximal isometric knee extensions with a 60-s rest between trials. Real-time visual feedback (Spike software v9.06; Cambridge Electronic Design, Cambridge, United Kingdom) and verbal encouragement were provided throughout all attempts. The highest isometric knee extension of the three recorded values was considered the MVC. To quantify the FS, force signals during the plateau phase of a sustained voluntary contraction performed at 25% MVC were low-pass filtered at 20 Hz (fourth-order Butterworth digital filter), and the coefficient of variation (CoV) of force was quantified.

### High-density surface electromyography (HDsEMG)

A semi-disposable high-density surface electromyography (HDsEMG) array (64 electrodes, 13 × 5, 8 mm, I.E.D., GR08MM1305, OT Bioelettronica, Inc., Turin, Italy) was positioned over the VL muscle belly with approximate orientation of the muscle fascicles (proximal to distal). Prior to this, the skin was prepared by shaving, applying light abrasion, and cleansing with 70% ethanol. These HDsEMG electrodes were secured using flexible tape and remained in place during the intervention and post-RE assessments. A ground electrode (WS2, OTBioelettronica, Turin, Italy) was positioned around the ankle of the tested leg. HDsEMG signals were acquired in a monopolar configuration, amplified (× 256) with filtering set at 10–500 Hz and digitally converted at 2000 Hz by a 16-bit wireless amplifier (Sessantaquattro, OTBioelettronica, Turin, Italy) and transferred to a PC for offline analysis.

### Intramuscular EMG (iEMG)

The motor point of VL was identified using low-intensity percutaneous electrical stimulations (400 V, pulse width 50 μS, current ∼10 mA; delivered via a Digitimer DS7 A, Welwyn Garden City, United Kingdom). The site of VL which produced the largest visible twitch from the smallest electrical current was considered the motor point and its proper localization is considered critical for electrode positioning [28].

Intramuscular electromyography (iEMG) recordings were obtained using a disposable concentric needle (model N53153, Teca, Hawthorne, New York, United States) with a recording area of 0.07 mm^2^. The grounding electrode was placed over the patella of the tested leg. Subsequently, the surrounding skin at the motor point was prepared by shaving and cleansing using an alcohol adhesive wipe. Participants were instructed to relax their tested leg to enable insertion of the needle into the muscle belly of VL until the appropriate depth of 1.0–2.5 cm was reached. Next, participants were instructed to perform isometric leg extension, and the signal quality was inspected, and the needle was adjusted where necessary. Subsequently, the needle was repositioned by rotating the bevel 180° and slightly retracted by ~ 5 mm to ensure that different depths of the muscle were captured [29]. Once the appropriate contraction sequence (sets of 25% of MVC) was completed (minimum of four recordings from distinct perspectives/regions), the needle was withdrawn and disposed of. The EMG signals were recorded at 50 kHz and bandpass filtered at 10 Hz to 10 kHz (CED Micro 1401; Cambridge Electronic Design, Cambridge, United Kingdom) and visualised using Spike2 (version 9, CED) software to provide real-time visual feedback on a screen positioned in front of the participants.

### Resistance exercise (RE) protocol

The acute RE protocol target was set as continuous sets of unilateral leg extension, with each set consisting of 12 repetitions and a 60-s rest period between the sets (Fig. 1 A). The dominant limb was engaged in RE on a standard leg extension machine (exercise leg), with the contralateral limb functioning as a control (control leg). The exercise intensity was set at the participant’s 75% of 1 RM and performed to failure. The criterion for failure was the participant’s inability to complete the full range of leg extension despite the investigator’s encouragement and was validated by the Borg rating of perceived exertion (RPE) scale corresponding to extremely hard-maximal exertion (19–20/20) [30]. The mean number of repetitions completed was 40 (SD; 3.06, range; 33–44), corresponding to 3 sets and 4 additional repetitions.

### Data analysis

The HDsEMG signals and corresponding force were selected from contraction number 3 or 4 of the 4 contractions performed. Each force trace was visually inspected and that with the least variation was chosen for analysis. This was analysed offline using the convolution kernel compensation method [31]. MU filters and corresponding spike trains estimated with this process [32] were visually inspected and optimised using custom software (DEMUSE, MATLAB) and standardised procedures, all performed by a single operator (MP) [33]. Only MUs with a pulse-to-noise ratio (PNR) ≥ 30 dB were included for further analyses. After editing each MU spike train, MUs were tracked from pre- to post-intervention via concatenation of signals and application of MU filters from one contraction (within each leg) to the other.

MU firing rate (MUFR) at the recruitment and derecruitment phases (Fig. 1E) of isometric knee extension was calculated as the mean instantaneous firing rate (FR) of the first 5 observations of each MU. MUFR during the sustained phase was the mean instantaneous FR throughout this segment. FR variability was considered as the CoV of the inter-spike interval (ISI) during the plateau phase. MU recruitment and derecruitment threshold was defined as the force level corresponding to the first and last observed firing of each MU, respectively. MUs that were tracked from pre- to post-intervention were used to compute a cumulative spike train (CST), which represents the neural drive to the muscle [34].

Decomposition of iEMG signals was performed using Decomposition-based quantitative electromyography (DQEMG) software, to extract motor unit potential trains (MUPTs) [35]. This includes preprocessing of signal, signal segmentation and MUP detection, clustering the identified MUPs, and supervised classification of detected MUPs [36]. Visual inspection of individual MUP templates was performed to ensure cursors were positioned accurately at the onset, endpoint, positive peak, and negative peak of the waveforms (Fig. 1D). Where required, cursors were adjusted and repositioned during visual inspection. MUPTs were excluded if they contained MUPs from multiple MUs or fewer than 40 MUPs [37].

MUP area, measured in µVms, was defined as the integral of the absolute value of the MUP values between the onset and end markers, multiplied by the sampling time interval [38]. MUP complexity was assessed using the number of turns in the MUP template, defined as the number of significant changes in direction within the MUP duration (height > 20 µv) [38]. The MUP negative peak ratio was calculated based on the absolute value of the ‘rise’ of the MUP template negative peak (within the 500 μs interval before the negative peak), divided by the ‘fall’ of the MUP template negative peak (within the 500 μs interval after the negative peak) [39]. Individual MUP templates were used to calculate a near-fibre MUP (NFM) [38]. Individual NFMs were visually inspected using DQEMG software, and any that were contaminated from other NFMs were excluded from the analysis. NFM parameters were used to estimate NF jiggle as an estimate of neuromuscular junction transmission instability [38].

### Statistical analysis

All statistical analysis was conducted using RStudio (Version 4.4.0). To assess differences in MVC, FS, HDsEMG, and iEMG MU variables, separate linear mixed-effects models were constructed with fixed effects of Time and Leg, and their interaction (Leg × Time). The model included random intercepts for each Subject to account for repeated measures. For tracked MU models, MUs were also nested within Subject. All linear-mixed effects models were generated using *lmer* R package [40]. Where significant main effects were detected, pairwise *post-hoc* tests of the estimated marginal means (EMMs) were performed [41]. To assess model fit and estimate the proportion of variance explained, the marginal R^2^ (R^2^ₘ) and conditional R^2^ (R^2^c) are included in each table of model outputs. The R^2^ₘ represents the proportion of variance explained by the fixed effects, and R^2^c represents the variance explained by both fixed effects and random effects [42]*.* Cohen’s d effect sizes are reported for all *post-hoc* comparisons. For non-hierarchical models (e.g., MVC and FS), effect sizes were calculated directly from paired data using the standard deviation of within-subject differences. For hierarchical models in which MUs were nested within subjects, Cohen’s d was calculated from EMMs derived from the linear mixed-effects models using *effectsize* R package [43], accounting for the nested data structure. Delta CST values were compared across limbs with a repeated measures t-test. Pearson correlation test was used to assess the strength, direction, and significance of the relationship between delta MVC and delta FR. The tabulated results are displayed as EMMs, 95% confidence intervals (CI), and *p* values. Statistical significance was accepted at *p* < 0.05.

## Results

### Participant characteristics

Participant characteristics are shown in Table 1. All participants completed the experimental session without any adverse effects.

**Table 1:** Descriptive characteristics of participants *(n* = *13; 5 female)*

Age (years)	74.9 (4.8)
Height (cm)	167.6 (6.8)
Weight (kg)	73.5 (11.7)
BMI (kg/m^2^)	26.1 (3.0)
VL CSA (cm^2^)	17.1 (4.3)

Data shown as mean (standard deviation). VL CSA obtained from the exercise (dominant) leg only at baseline. *Abbreviations*:* BMI *body mass index,* VL CSA *vastus lateralis cross-sectional area

### Functional properties

**Fig. 2:**
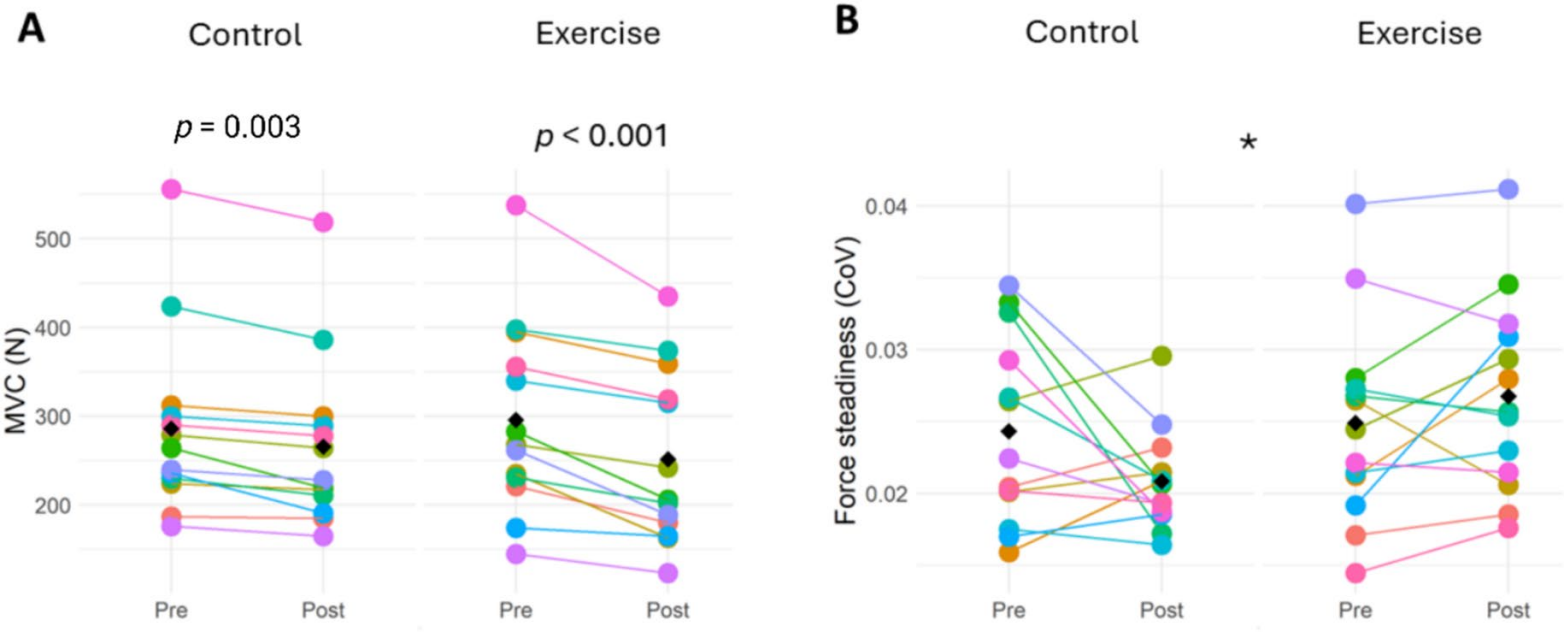
Effect of acute unilateral RE on knee extensor MVC (A) and force steadiness (B) in control (left) and exercise (right) legs. Data points are colour-coded for individual participants with the mean of means indicated by black diamonds. *Significant difference between the exercise and control legs at post-intervention. *Abbreviations*: *MVC *maximal voluntary contraction,* CoV *Coefficient of variation of force at 25% of MVC

All model outputs including estimated marginal means (EMM) for functional properties are shown in Table 2. Knee extensor MVC at each timepoint (pre and post) for the exercise (right) and control (left) legs for 13 participants are shown in Fig. 2A. There was a significant leg × time interaction on knee extensor MVC (*p* = 0.013), and a main effect of time (*p* = 0.003), with no main effect of leg (*p* = 0.388). EMMs show a mean decrease in MVC of 14.8% (*p* < 0.001, *d* = 1.58) in the exercise leg, and of 6.9% in the control leg (*p* = 0.003, *d* = 1.36).

**Table 2:** Summary of linear regression analyses for functional measures and HDsEMG non-tracked MUs

	Pre-intervention		Post-intervention		Model fit	Leg	Time	Leg × Time
	Control	Exercise	Control	Exercise				
***Functional assessments***								
MVC (N)	286 (226–346)	296 (235–356)	266 (205–326)	252 (191–312)	R^2^ₘ = 0.03 R^2^c = 0.98	0.388	0.003*	0.013*
Force steadiness (CoV)	0.024 (0.020–0.028)	0.025 (0.021–0.029)	0.021 (0.018–0.024)	0.027 (0.024–0.030)	R^2^ₘ = 0.11 R^2^c = 0.61	0.742	0.062	0.032*
***HDsEMG non-tracked MU***								
No of MUs	177	178	133	128				
FR recruitment (pps)	6.21 (5.76–6.66)	5.89 (5.44–6.34)	6.01 (5.55–6.48)	6.69 (6.23–7.16)	R^2^ₘ = 0.04 R^2^c = 0.25	0.001*	0.182	0.001*
FR plateau (pps)	7.43 (6.75–8.11)	7.32 (6.65–8.00)	7.26 (6.57–7.95)	8.66 (7.97–9.35)	R^2^ₘ = 0.09 R^2^c = 0.42	0.001*	0.280	0.001*
FR derecruitment (pps)	5.04 (4.62–5.46)	5.09 (4.67–5.51)	5.12 (4.69–5.55)	6.07 (5.64–6.50)	R^2^ₘ = 0.09 R^2^c = 0.35	0.001*	0.467	0.001*
FR variability (%)	0.141 (0.127–0.155)	0.124 (0.110–0.138)	0.137 (0.121–0.153)	0.112 (0.096–0.128)	R^2^ₘ = 0.03 R^2^c = 0.06	0.607	0.619	0.476
Recruitment threshold (N)	33.3 (24.8–41.9)	33.6 (25.1–42.2)	35.6 (26.9–44.3)	31.8 (23.1–40.5)	R^2^ₘ = 0.01 R^2^c = 0.34	0.331	0.277	0.176
Derecruitment threshold (N)	30.9 (22.8–39.0)	34.5 (26.4–42.6)	31.1 (22.9–39.3)	35.3 (27.1–43.5)	R^2^ₘ = 0.01 R^2^c = 0.42	0.441	0.906	0.810

Data presented as estimated marginal means (lower 95% CI, upper 95% CI). Final four columns show model fit and *p* values. *Abbreviations*: *MVC *maximal voluntary contraction,* CoV *Coefficient of variation of force at 25% of MVC,* FR *firing rate*,* R^2^ₘ, marginal R^2^ representing variance explained by fixed effects only; R^2^c, conditional R^2^ representing variance explained by the full model (fixed + random effects). *Statistically significant (*p* < 0.05)

For FS, there was a significant leg × time interaction (*p* = 0.032), but no effect of leg (*p* = 0.742) or time (*p* = 0.062). *Post-hoc* analysis indicated that FS CoV was comparable between the legs at baseline (*p* = 0.743, *d* = 0.093), however, following the intervention, FS CoV was higher in the exercise leg compared to the control leg *(p* = 0.002, *d* = 0.889) (Fig. 2B).

### Motor unit discharge characteristics

From HDsEMG, a total of 616 MUs from 13 participants were sampled in both limbs at both timepoints. Individual MU counts and all model outputs are shown in Table 2.

**Fig. 3:**
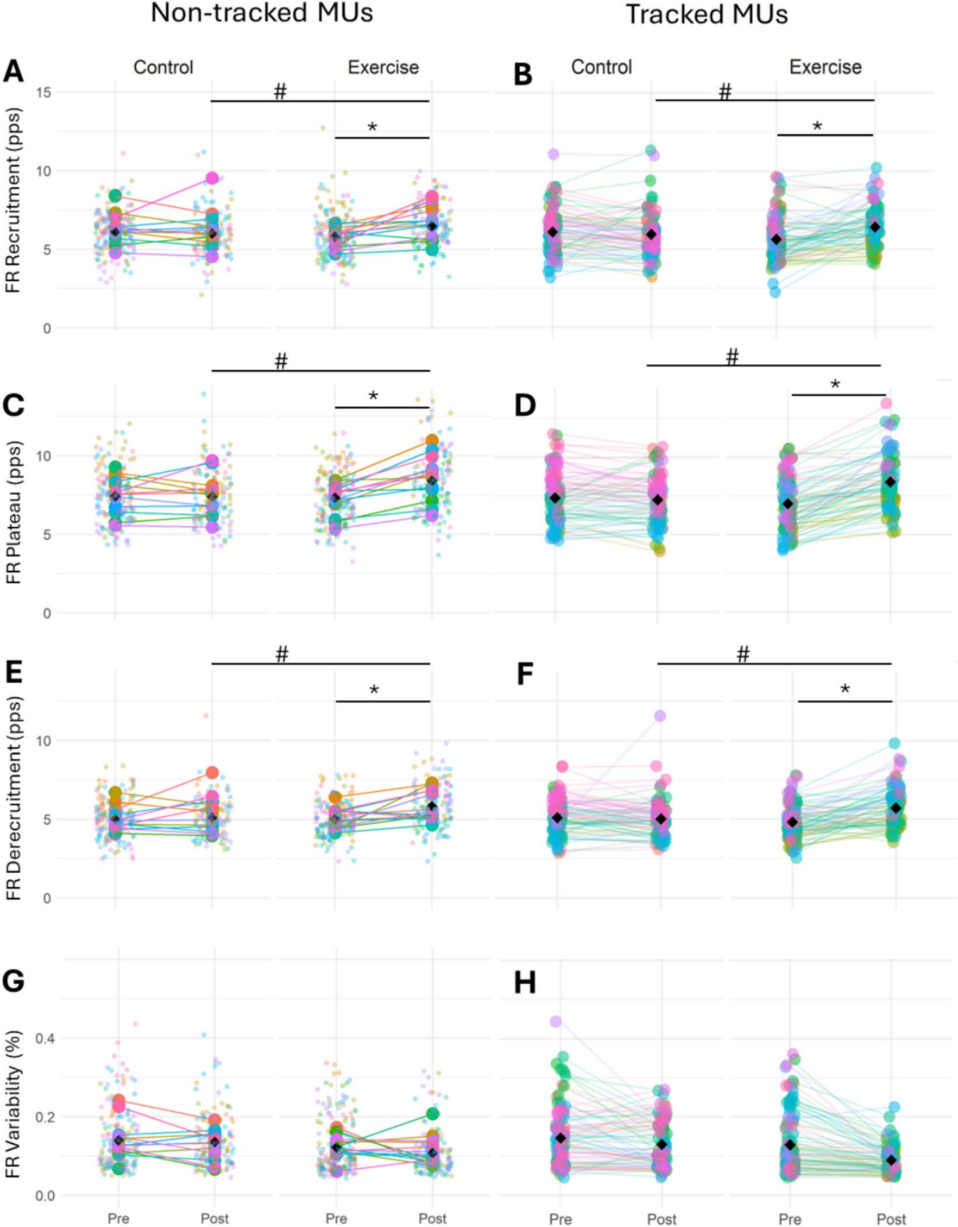
Motor unit firing rate (MUFR) at 25% MVC for non-tracked and tracked motor units in the exercise and control legs before and after the unilateral RE intervention. MUFR at the recruitment phase for non-tracked (**A**) and tracked (**B**) motor units. MUFR at plateau phase for non-tracked (**C**) and tracked (**D**) motor units. MUFR at derecruitment phase for non-tracked (**E**) and tracked (**F**) motor units. MUFR variability for non-tracked (**G**) and tracked (**H**) motor units during the plateau phase. Data points are colour-coded for individual participants with the mean of means indicated by black diamonds. Statistical analyses are based on multi-level mixed effects linear models. *Significantly different between pre and post timepoints (*p* < 0.001). ^#^ Significant difference between the exercise and control legs at post intervention (*p* < 0.001). *Abbreviations*:* FR *firing rate,* MUs *motor units

MU firing rate (MUFR) at recruitment showed a significant leg × time interaction (*p* = 0.001), a significant effect of leg (*p* = 0.001), but no effect of time (*p* = 0.182). EMMs showed that, at baseline, MUFR at recruitment was greater in the control leg compared to the exercise leg (*p* = 0.02, *d* = 0.171), however post-intervention, it was greater in the exercise leg (*p* < 0.001, *d* = 0.369). Moreover, MUFR at recruitment did not differ in the control leg from pre- to post-intervention (*p* = 0.182, *d* = 0.105), but for the exercise leg, MUFR at recruitment was significantly greater at post compared to baseline (*p* < 0.001, *d* = 0.435) (Table 2, Fig. 3A).

During the plateau phase, there was a significant leg × time interaction (*p* < 0.001) and a significant effect of leg (*p* < 0.001), but there was no main effect of time (*p* = 0.280). While MUFR at plateau was similar between the legs at baseline (*p* = 0.477, *d* = 0.056), following the intervention, FR at plateau was greater for the exercise leg compared to the control leg (*p* < 0.001, *d* = 0.741). Additionally, while MUFR at plateau did not change in the control leg across timepoints (*p* = 0.280, *d* = 0.091), it was significantly greater following the intervention compared to the baseline for the exercise leg *(p* < 0.001, *d* = 0.705) (Table 2, Fig. 3C).

For MUFR at derecruitment, there was a significant leg × time interaction (*p* < 0.001) and a significant effect of leg (*p* < 0.001), but there was no significant effect of time (*p* = 0.467). EMMs showed that MUFR at derecruitment was comparable between legs at baseline (*p* = 0.622, *d* = 0.032), but following the intervention, MUFR at derecruitment was greater for the exercise leg compared to the control leg (*p* < 0.001, *d* = 0.547). Additionally, although MUFR at derecruitment did not change in the control leg from baseline to post (*p* = 0.466, *d* = 0.050), there was greater MUFR at derecruitment at post compared to baseline for the exercise leg (*p* < 0.001, *d* = 0.565) (Table 2, Fig. 3E).

There was no significant leg × time interaction for MUFR variability (*p* = 0.476) and no significant effect of leg (*p* = 0.607), or time (*p* = 0.619) (Table 2, Fig. 3G).

**Fig. 4:**
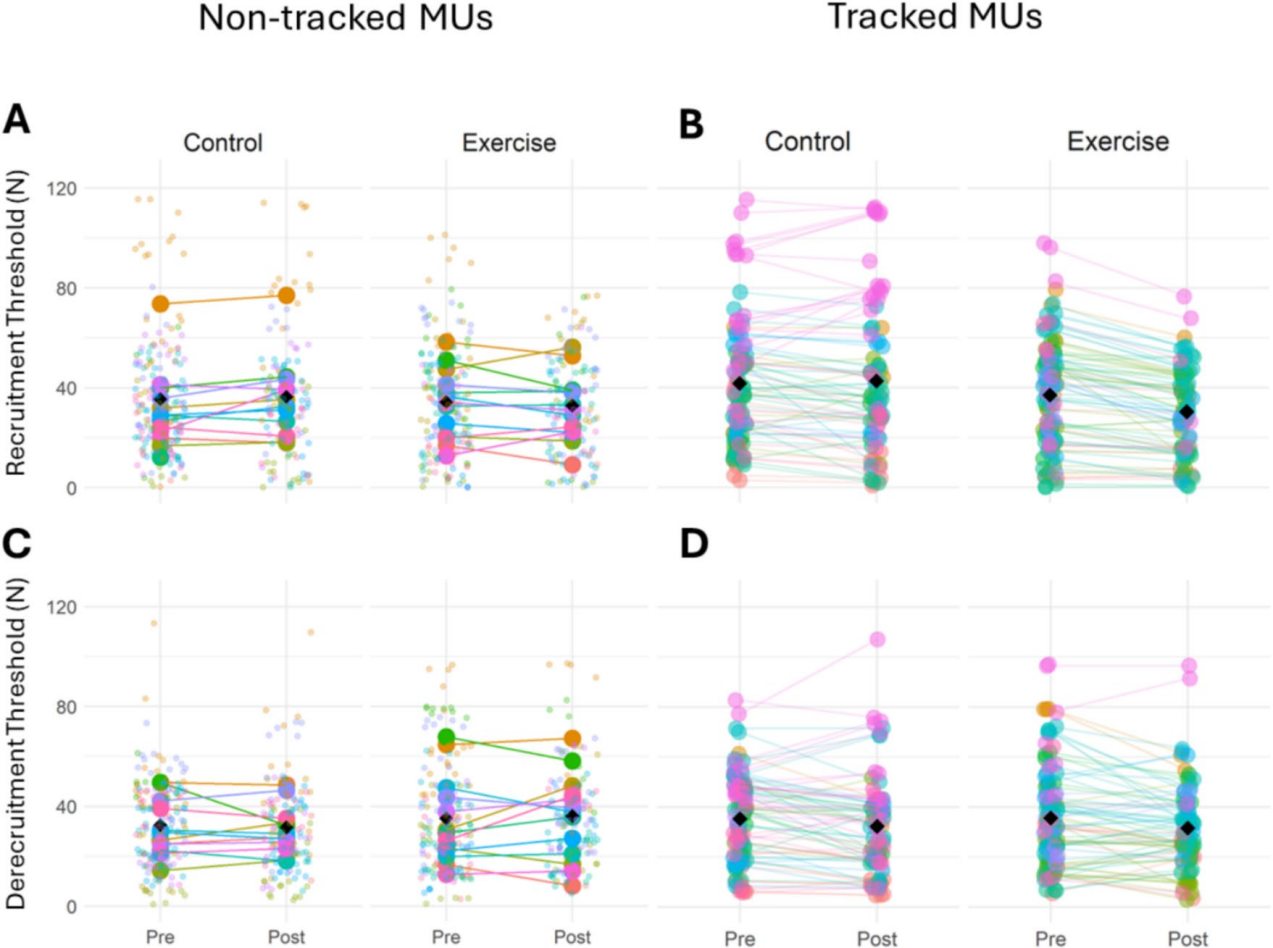
Motor unit recruitment and derecruitment thresholds at 25% MVC for non-tracked and tracked motor units in the exercise and control legs before and after the unilateral RE intervention. MU recruitment threshold for non-tracked (**A**) and tracked (**B**) motor units. MU derecruitment threshold for non-tracked (**C**) and tracked (**D**) motor units. Data points are colour-coded for individual participants with the mean of means indicated by black diamonds. Statistical analyses are based on multi-level mixed effects linear models

For MU recruitment threshold, there was no significant leg × time interaction (*p* = 0.176), or significant effect of leg (*p* = 0.331) or time (*p* = 0.277) (Table 2, Fig. 4A). Similarly, for MU derecruitment threshold, there were no significant leg × time interaction (*p* = 0.810), or significant effect of leg (*p* = 0.441), or time (*p* = 0.906) (Table 2, Fig. 4C).

### Discharge characteristics of tracked MUs

A total of 374 MUs (61% of all recorded MUs) from 13 participants were successfully tracked from pre- to post-intervention: 179 in the control leg and 195 in the exercised leg. All model outputs for tracked MUs are shown in Table 3.

**Table 3:** Summary of linear regression analyses for HDsEMG tracked MUs

	Pre-intervention		Post-intervention		Model fit	Leg	Time	Leg × Time
	Control	Exercise	Control	Exercise				
FR recruitment (pps)	6.28 (5.66–6.90)	5.82 (5.21–6.43)	6.17 (5.54–6.80)	6.76 (6.13–7.39)	R^2^ₘ = 0.06 R^2^c = 0.51	0.008*	0.545	0.001*
FR plateau (pps)	7.33 (6.57–8.08)	7.12 (6.37–7.86)	7.16 (6.40–7.93)	8.69 (7.93–9.46)	R^2^ₘ = 0.14 R^2^c = 0.68	0.257	0.408	0.001*
FR derecruitment (pps)	5.14 (4.65–5.63)	5.00 (4.52–5.48)	5.06 (4.56–5.56)	6.05 (5.55–6.55)	R^2^ₘ = 0.14 R^2^c = 0.73	0.303	0.576	0.001*
FR Variability (%)	0.148 (0.130–0.168)	0.133 (0.115–0.152)	0.134 (0.114–0.155)	0.097 (0.077–0.117)	R^2^ₘ = 0.08 R^2^c = 0.49	0.095	0.152	0.110
Recruitment threshold (N)	39.5 (28.9–50.2)	38.5 (28.0–49.0)	38.8 (28.1–49.6)	33.3 (22.6–44.1)	R^2^ₘ = 0.12 R^2^c = 0.69	0.688	0.800	0.222
Derecruitment threshold (N)	34.7 (26.5–43.0)	37.3 (29.1–45.5)	31.4 (23.0–39.9)	34.7 (26.3–43.1)	R^2^ₘ = 0.15 R^2^c = 0.55	0.223	0.142	0.839

Data presented as estimated marginal means (lower 95% CI, upper 95% CI). Final four columns show model fit and *p* values. *Abbreviations*: *FR *firing rate, *R*^*2*^*ₘ* marginal R^2^ representing variance explained by fixed effects only, *R*^*2*^*c*, conditional R^2^ representing variance explained by the full model (fixed + random effects). *Statistically significant (*p* < 0.05)

For MUFR at recruitment, there was a significant leg × time interaction (*p* < 0.001) and a significant effect of leg (*p* = 0.008), but there was no effect of time (*p* = 0.545). EMMs analysis indicated that at baseline, MUFR at recruitment was higher in the control leg compared to the exercise leg (*p* = 0.008, *d* = 0.265), whereas post-intervention, MUFR at recruitment was higher in the exercise leg compared to the control leg (*p* = 0.003, *d* = 0.303). Additionally, MUFR at recruitment did not differ in the control leg from pre to post (*p* = 0.545, *d* = 0.045), but for the exercise leg, it was significantly greater at post compared to the baseline (*p* < 0.001, *d* = 0.613) (Table 3, Fig. 3B).

There was a significant leg × time interaction for MUFR at the plateau (*p* < 0.001), but there was no effect of leg (*p* = 0.257) or time (*p* = 0.408). EMMs analysis showed that MUFR at the plateau was comparable between the legs at baseline (*p* = 0.257, *d* = 0.122), but post-intervention, it was greater in the exercise leg compared to the control leg (*p* < 0.001, *d* = 0.818). Moreover, although MUFR at the plateau did not differ in the control leg across timepoints (*p* = 0.408, *d* = 0.043), MUFR at the plateau significantly increased from baseline to post in the exercise leg (*p* < 0.001, *d* = 0.982) (Table 3, Fig. 3D).

Considering MUFR at derecruitment, there was a significant leg × time interaction (*p* < 0.001), but there was no difference between the legs (*p* = 0.303) or timepoints (*p* = 0.510). EMMs analysis indicated that MUFR at derecruitment did not differ between the legs at baseline (*p* = 0.303, *d* = 0.076), but following the intervention, MUFR at derecruitment was significantly greater for the exercise leg compared to the control leg (*p* < 0.001, *d* = 0.588). Additionally, MUFR at derecruitment in the control leg did not change across timepoints (*p* = 0.576, *d* = 0.298), but for the exercise leg, MUFR at derecruitment was significantly greater at post compared to the baseline (*p* < 0.001, *d* = 0.963) (Table 3, Fig. 3F).

Considering FR variability, there was no significant interaction between leg × time (*p* = 0.110) and no significant differences between legs (*p* = 0.095) nor timepoints (*p* = 0.152) (Table 3, Fig. 3H).

For the recruitment threshold, there was no leg × time interaction (*p* = 0.222), or significant effect of leg (*p* = 0.688), or time (*p* = 0.800) (Table 3, Fig. 4B). Similarly, for derecruitment threshold, there was no leg × time interaction (*p* = 0.839), or significant effect of leg (*p* = 0.223), or time (*p* = 0.839) (Table 3, Fig. 4D).

### Cumulative spike train

**Fig. 5:**
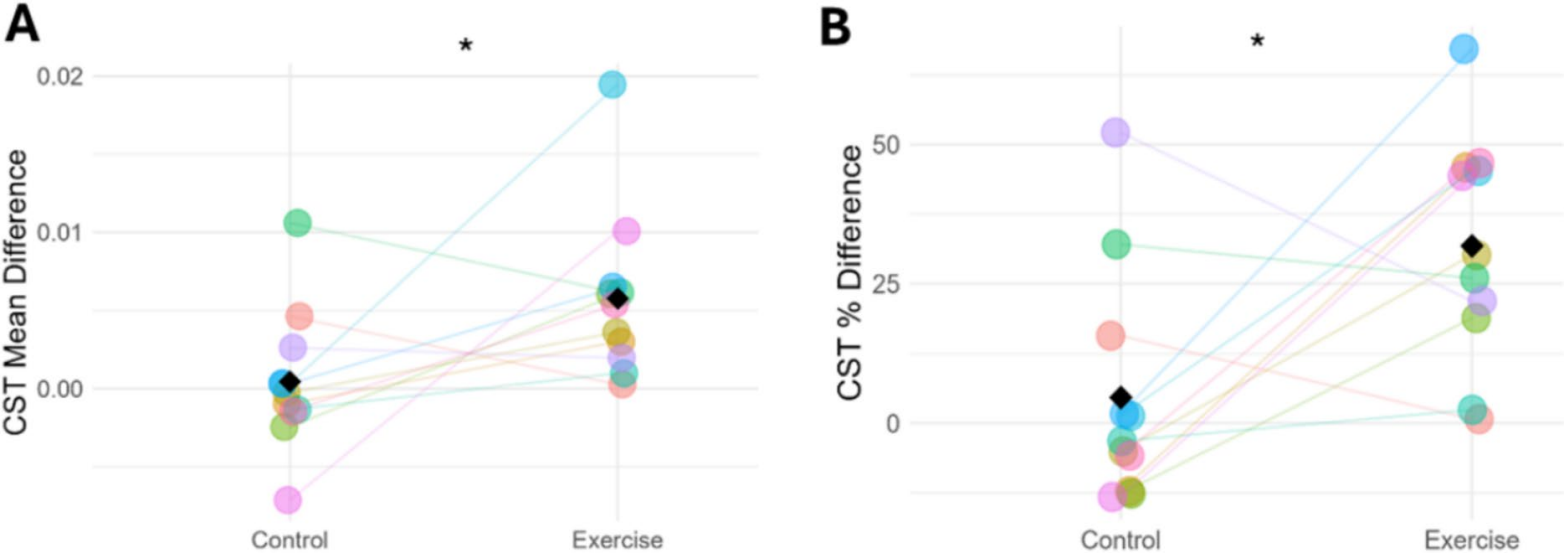
The difference from pre- to post-intervention of the cumulative spike train shown as an absolute value (**A**) and as a percentage (**B**). Data points are colour-coded for individual participants with means indicated by black diamonds. *Significantly different between the exercise and control legs (*p* < 0.05). *Abbreviations*:* CST *cumulative spike train

MUs that were tracked from pre- to post-intervention were used to compute the cumulative spike train (CST) for each leg at each timepoint. The pre to post difference in the mean of the CST was greater in the exercised than the control leg (*p* = 0.043), and the same was true of the percentage difference (*p* = 0.022) (Fig. 5).

### Correlation between changes in MUFR and MVC

**Fig. 6:**
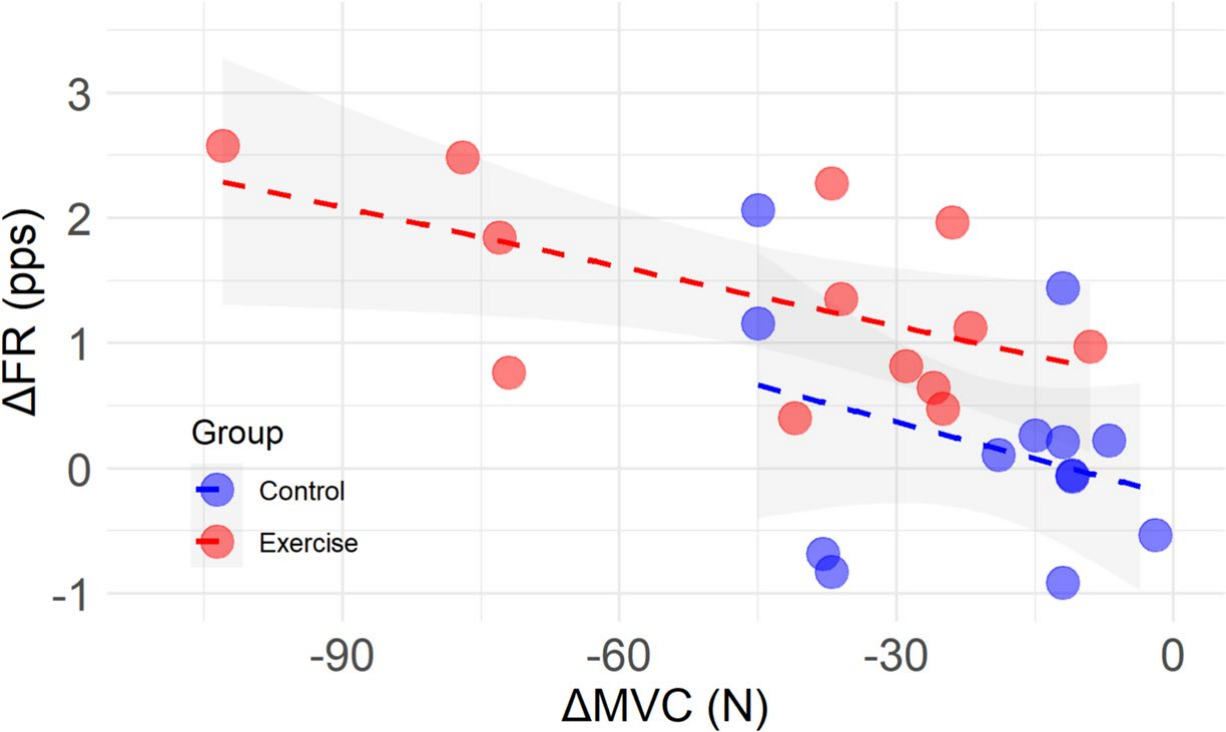
Correlation of change in firing rate as a function of change in MVC, from pre- to post-intervention in the control (blue) and exercise (red) legs. *Abbreviations*:* FR *firing rate,* pps *pulses per second,* MVC *maximal voluntary contraction

There was a weak yet statistically significant correlation between delta FR and delta MVC in the exercise leg (*r* = −0.566, *p* = 0.043), which was not observed in the control leg (*r* = −0.330, *p* = 0.271) (Fig. 6).

### iEMG-derived motor unit characteristics

A total of 841 MUPs were recorded from both legs at both time points for 11 participants (4 females). All Individual MU counts and model outputs for iEMG data are shown in Table 4.

**Table 4:** Summary of linear regression analyses for iEMG motor unit properties

	Pre-intervention		Post-intervention		Model fit	Leg	Time	Leg × Time
	Control	Exercise	Control	Exercise				
No of MUPs	306	238	142	155				
MUP Area (µV.ms)	828 (744—913)	901 (812—991)	781 (675—886)	796 (694—899)	R^2^ₘ = 0.01 R^2^c = 0.06	0.119	0.392	0.461
MUP Turns	4.42 (4.03—4.82)	4.53 (4.12—4.93)	4.60 (4.16—5.04)	4.61 (4.17—5.05)	R^2^ₘ = 0.03 R^2^c = 0.12	0.503	0.332	0.714
NF Jiggle (%)	17.4 (14.8—19.9)	18.0 (15.4—20.6)	17.8 (15.0—20.6)	18.0 (15.2—20.7)	R^2^ₘ = 0.02 R^2^c = 0.21	0.472	0.673	0.738
Negative peak ratio	2.01 (1.72—2.29)	1.99 (1.69—2.29)	2.24 (1.90—2.58)	1.89 (1.55—2.22)	R^2^ₘ = 0.04 R^2^c = 0.04	0.896	0.168	0.164

Data presented as estimated marginal means [lower 95% CI, upper 95% CI]. Final four columns show model fit and *p* values. *Abbreviations*: *MUP* motor unit potential,*NF* near fibre, *R*^*2*^*ₘ* marginal R^2^ representing variance explained by fixed effects only, *R*^*2*^*c* conditional R^2^ representing variance explained by the full model (fixed + random effects)

**Fig. 7:**
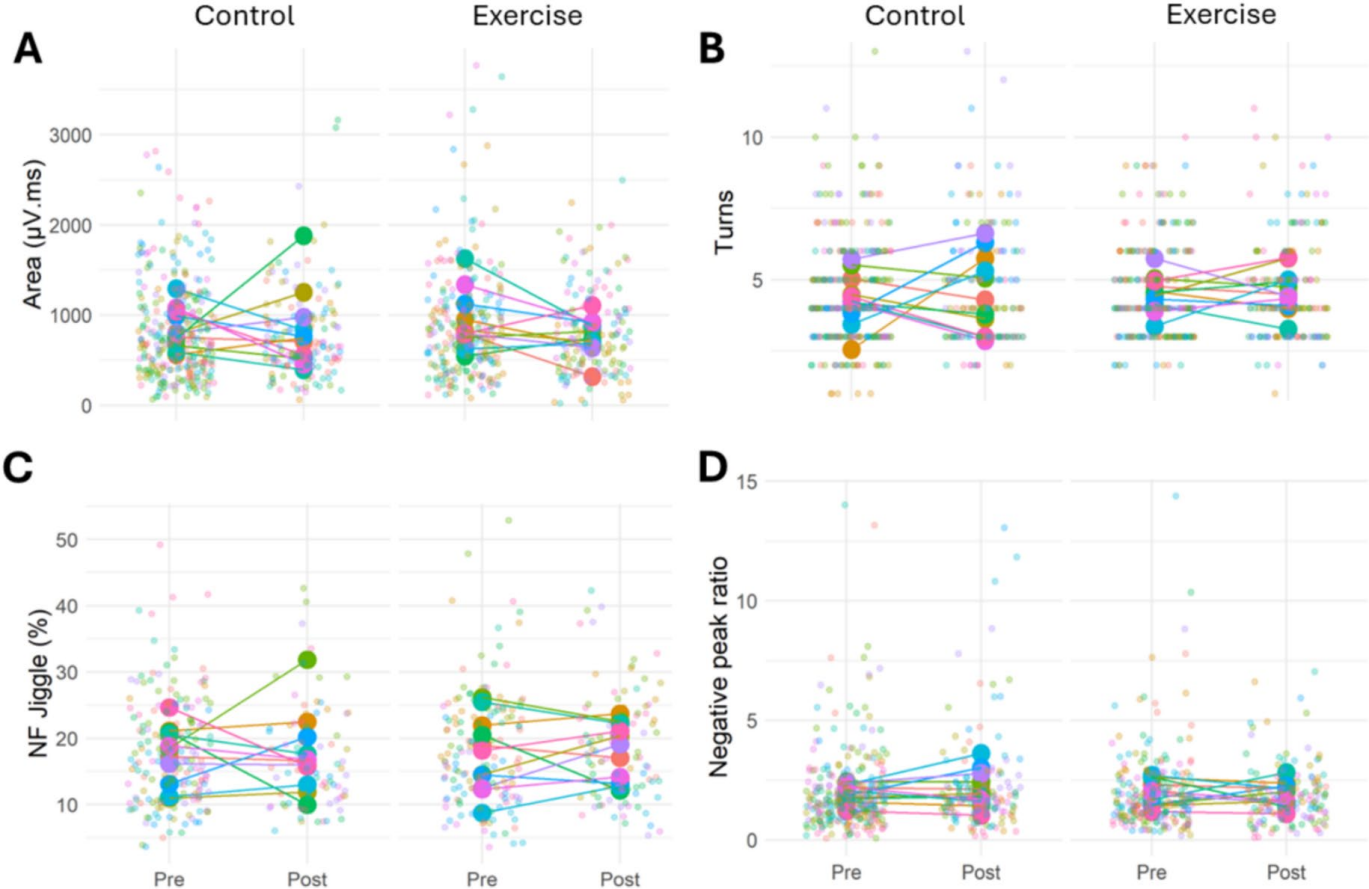
Motor unit characteristics assessed using iEMG. Motor unit Area (**A**), number of turns (**B**), neuromuscular junction transmission instability (Near fibre jiggle) (**C**), and negative peak ratio (**D**) in the exercise and control legs before and after the unilateral RE intervention. Data points are colour-coded for individual participants. Statistical analyses are based on multi-level mixed effects linear models

For MUP area, there were no leg × time interaction (*p* = 0.461), or significant effects of leg (*p* = 0.119), or time (*p* = 0.392). Similarly, for MUP turns there were no significant leg × time interaction (*p* = 0.714) or effects of leg (*p* = 0.503), or time (*p* = 0.332). For NF jiggle, there was no leg × time interaction (*p* = 0.738), or significant effects of leg (*p* = 0.472), or time (*p* = 0.673). Additionally, for the negative peak ratio, there were no significant leg × time interaction (*p* = 0.164), or effects of leg (*p* = 0.896), or time (*p* = 0.168) (Table 4, Fig. 7).

## Discussion

The present study evaluated the effect of acute unilateral knee extensor RE on bilateral neuromuscular function and VL MU features in healthy older adults. The main findings indicate that a single bout of unilateral resistance exercise is sufficient to elicit reductions in maximal knee extensor voluntary force in the exercised and to a lesser extent, in the contralateral non-exercised limb, demonstrating a cross-limb transfer of muscle performance fatigue. However, the majority of markers of individual MU function were altered only in the exercise leg, with MUFR increasing during all phases of a contraction normalised to 25% of baseline MVC, with no notable adaptation in the non-exercise limb.

The resistance-based exercise used in this study was deliberately selected to induce performance fatigue in VL which plays a crucial role in daily activities. Resistance exercise-induced reduction in force of ~ 15% in the exercise limb and ~ 7% in the non-exercise limb in the current study supports previous findings in young adults [21–23, 44]. Similarly, impaired force accuracy was only apparent in the exercised limb, supportive of previous fatigue-related findings in young adults [45].

The decline in contractile function leading to reduced MVC in the exercise leg is undoubtedly partly caused by molecular mechanisms (e.g. reduced sarcoplasmic reticulum Ca^2+^ release, impaired myofibrillar force production, and reduced myofibrillar Ca^2+^ sensitivity [46, 47]), and the increase in MUFR reported here, which correlated with declines in MVC, likely occurred to compensate for this. Indeed, the loss of force in the exercised leg was twofold greater than the control. However, mechanisms explaining the loss in the control leg are less clear.

Earlier studies of cross-limb transfer showed voluntary activation of plantar flexors was reduced following eccentric contractions, in the exercised and contralateral non-exercised limb [48], and separately, sustained fatigue protocols impaired voluntary activation bilaterally following unilateral quadriceps exercise [49]. Both of these suggest a strong central component of this phenomenon, although unlikely to be mediated by group III/IV afferents [50]. However, we found no alteration of MUFR in the control limb from pre- to post- intervention, albeit when sampled at 25% MVC. Moreover, when estimating neural drive via the CST calculated from the firing times of MUs tracked pre to post, this also showed no alteration in the control limb. This limits the plausibility of the previous hypothesis of diminished neural drive as a causal factor to contralateral decrements. Although sampled in submaximal contractions in the current study, these were normalised to baseline MVC and therefore represent a higher *relative* force value but cannot be extrapolated across the higher recruitment range.

Non-local muscle fatigue may also be mediated by the circulation of metabolic by-products generated within the fatigued muscles [51]. The accumulation of metabolites such as potassium, hydrogen, heat-shock proteins, and blood lactate [51, 52] in the exercised muscle can migrate and exert non-local fatigue effects that impair enzymatic activity, muscle contractile dynamics, and action potential transmission [53, 54]. Additionally, repeated muscle activation is known to elevate extracellular potassium levels [55], which may migrate into non-exercise muscles. This repeated muscle activation alters the electrochemical gradients for potassium, which could contribute to diminished force production [56] and reduced excitability of the muscle, thereby contributing to performance fatigue [57].

The current study found no change in MUFR variability at 25% MVC in either leg, which corresponds to previous studies with eccentric and concentric fatigue protocols [39]. Additionally, we found no changes in MU recruitment and derecruitment thresholds in either leg, including of MUs tracked across the intervention. However, previous studies have shown increased [58] and decreased [59] recruitment thresholds. These disparities in findings could be attributed to variations in task, muscle, and age groups considered.

The concurrent use of iEMG enabled a more detailed assessment of peripheral MU characteristics, and we found no RE-induced changes in MU properties in the exercise or contralateral control leg. The MUP negative peak ratio quantifies the relationship between the rise and fall slopes of the negative peak of the MUP template and may be sensitive to metabolite accumulation. In healthy young, this was altered following concentric fatigue protocols (lasting ~ 1 h), but not eccentric [39]. These discrepancies could be primarily attributed to the variations in exercise protocols, particularly exercise duration. The NF jiggle is an indicator of NMJ transmission instability, which shows the variability of temporal dispersion across MU action potential [38] and is mainly related to the activity of muscle fibres close to the detection surface of the concentric needle electrode (within ~ 350 µm) [60]. Hence, the contributions of distant fibres to NF MUPs are minimal, which reduces the contamination from other MUs. However, although eccentric and concentric exercise protocols in healthy young individuals have been shown to increase NF jiggle [39], our resistance exercise protocol resulted in no change in NF jiggle in either leg. Again, it is probable these differences are explained by the shorter duration of the RE in the current study.

### Limitations

Firstly, the current study focused exclusively on immediate neuromuscular adaptations following a single bout of unilateral resistance exercise-induced performance fatigue. Therefore, these findings may not generalize to other types of intervention, such as those targeting endurance adaptations. Secondly, the current study reported MU data recruited at lower contraction levels (25% MVC), while the exercise intervention was performed at a higher intensity (75% of 1RM). The MU assessment at higher contraction intensities was restricted by intramuscular needle placement. Therefore, the current study results may not be applicable for MUs with higher recruitment thresholds which are being recruited at higher force levels. Additionally, given the previously reported duration-dependent changes in muscle fibre conduction velocity in VL [61], post-intervention measures may be influenced by transient recovery which is an inherent limitation of this study design. To minimize potential biases, testing between the legs was randomised at all timepoints. Moreover, this study did not include a healthy young cohort, so it was not possible to infer any age-related alterations in neuromuscular adaptations. Finally, the current study was unable to determine the specific mechanism/s underlying the reduction in force in the control limb. Future studies could include neurophysiological measures such as transcranial magnetic stimulation to further explore the mechanisms underlying the acute effects of cross-limb transfer.

## Conclusions

The current study demonstrates that a single bout of unilateral knee extensor resistance exercise performed to fatigue is sufficient to elicit a bilateral reduction in maximal forces with neural adaptations occurring only in the exercise limb. These findings indicate that resistance exercise-induced muscle fatigue results in immediate central and peripheral alterations in the exercise limb. While the exact mechanism accounting for the force decline in the non-exercise limb remains uncertain, it is mainly suggestive of adaptations at systemic and/or central levels. These findings are important in understanding neurophysiological mechanisms following resistance exercise-based performance fatigue and may have potential implications for clinical settings including those often experienced by older adults (e.g. single-leg fracture, post-injury immobilisation, and stroke).

## Data Availability

The datasets generated and analysed during the current study are available from the corresponding author upon reasonable request.
